# Ethical and practical considerations for HIV cure-related research at the end-of-life: a qualitative interview and focus group study in the United States

**DOI:** 10.1186/s12910-022-00741-8

**Published:** 2022-01-11

**Authors:** John Kanazawa, Sara Gianella, Susanna Concha-Garcia, Jeff Taylor, Andy Kaytes, Christopher Christensen, Hursch Patel, Samuel Ndukwe, Stephen A. Rawlings, Steven Hendrickx, Susan Little, Brandon Brown, Davey Smith, Karine Dubé

**Affiliations:** 1 Gillings School of Global Public Health, University of North Carolina at Chapel Hill, 4108 McGavran-Greenberg Hall, Chapel Hill, NC USA; 2 Department of Medicine, Division of Infectious Diseases and Global Public Health, University of California San Diego, La Jolla, CA USA; 3 AntiViral Research Center (AVRC), University of California at San Diego, 220 Dickinson Street, Suite A, San Diego, CA USA; 4 HIV Neurobehavioral Research Program (HNRP), California NeuroAIDS Tissue Network, University of California San Diego, 220 Dickson Street, Suite B, San Diego, CA USA; 5 AVRC Community Advisory Board, University of California San Diego, 220 Dickinson Street, Suite A, San Diego, CA USA; 6 HIV + Aging Research Project – Palm Springs (HARP-PS), 1775 Palm Canyon Drive, Suite 110-349, Palm Springs, CA USA; 7 Department of Social Medicine, Population and Public Health, Center for Healthy Communities, University of California, Riverside, 3333 14th Street, Riverside, CA USA

**Keywords:** HIV cure research, Last Gift, Rapid research autopsy, End-of-life, Altruism, Empirical ethics, People with HIV

## Abstract

**Background:**

One of the next frontiers in HIV research is focused on finding a cure. A new priority includes people with HIV (PWH) with non-AIDS terminal illnesses who are willing to donate their bodies at the end-of-life (EOL) to advance the search towards an HIV cure. We endeavored to understand perceptions of this research and to identify ethical and practical considerations relevant to implementing it.

**Methods:**

We conducted 20 in-depth interviews and 3 virtual focus groups among four types of key stakeholders in the United States (PWH, biomedical HIV cure researchers, HIV clinicians, and bioethicists) to obtain triangulated viewpoints because little was known about the ethics of this topic. Each group was queried as to ethical considerations, safeguards, and protections for conducting HIV cure-related research at the EOL to ensure this research remains acceptable.

**Results:**

All four key stakeholder groups generally supported HIV cure-related research conducted at the EOL because of the history of altruism within the PWH community and the potential for substantial scientific knowledge to be gained. Our informants expressed that: (1) Strong stakeholder and community involvement are integral to the ethical and effective implementation, as well as the social acceptability of this research; (2) PWH approaching the EOL should not inherently be considered a vulnerable class and their autonomy must be respected when choosing to participate in HIV cure-related research at the EOL; (3) Greater diversity among study participants, as well as multi-disciplinary research teams, is necessitated by HIV cure-related research at the EOL; (4) The sensitive nature of this research warrants robust oversight to ensure a favorable risk/benefit balance and to minimize the possibility of therapeutic misconception or undue influence; and (5) Research protocols should remain flexible to accommodate participants’ comfort and needs at the EOL.

**Conclusion:**

Because of the ethical issues presented by HIV cure-related research at the EOL, robust ethical safeguards are of utmost importance. The proposed ethical and practical considerations presented herein is a first step in determining the best way to maximize this research’s impact and social value. More much inquiry will need to be directed towards understanding context-specific and cultural considerations for implementing EOL HIV cure research in diverse settings.

**Supplementary Information:**

The online version contains supplementary material available at 10.1186/s12910-022-00741-8.

## Background

HIV/AIDS is no longer considered a terminal illness as it was in the 1980s. Instead, it is now a manageable chronic condition due in large part to significant advances in antiretroviral therapy (ART) over the past four decades [1–3]. People with HIV (PWH) who are adherent to ART have a life expectancy near that of the general population [4, 5]. Yet despite highly potent and effective ART, UNAIDS estimated that approximately 41% of PWH remain unsuppressed among countries with reportable data in 2019 [6]. One of the next frontiers in HIV research is focused on finding a cure, a long hoped-for but previously unascertainable goal [7]. Currently, there have been over 250 completed or active biomedical studies related to HIV cure worldwide [8]. Most HIV cure-related studies to date have enrolled otherwise healthy PWH [3, 9]. A new priority now includes extremely altruistic PWH with non-AIDS terminal illnesses who are willing to donate their bodies at the end-of-life (EOL) to advance the search towards an HIV cure [2, 3]. This type of research is already occurring in the United States and Canada [10, 11] and potentially expanding to other global settings as well.

The purpose of HIV cure-related research at the EOL is to characterize and quantify the latent HIV reservoir, a task for which large deep-tissue samples are required [3, 10]. Rapid research autopsies are currently the only feasible method for obtaining such samples since the HIV genome rapidly begins to degrade soon after death [10–12]. The rationale for conducting HIV cure-related or persistence research in PWH at the EOL is six-fold: (1) there is no reasonable expectation of direct clinical benefits associated with these studies, (2) the community of aging PWH has expressed a manifest desire to advance HIV cure-related research [2], (3) limited opportunities exist for terminally ill PWH to participate in any kind of HIV clinical research, (4) people near the EOL may be willing to accept higher risks for research participation, (5) rapid research autopsy is possible in this population through body donation, and (6) a unique opportunity is presented to create a novel translational model to test interventions on human participants [3].

The Last Gift Study is one such observational research study conducted at the University of California San Diego (UCSD) that enrolls terminally ill volunteers who have a prognosis of six months or less, as well as chronically-ill PWH with multiple co-morbidities towards the EOL [3]. Enrolled participants in the Last Gift Study voluntarily agree to donate blood and other samples ante-mortem and their bodies post-mortem to advance HIV cure-related science [3]. Enrolling in a research study at the EOL and donating one’s body for rapid research autopsy is rich with ethical dilemmas [12–14], yet the potential scientific knowledge generated may be significant [15, 16]. Our manuscript builds upon our prior work which details normative ethical considerations for observational HIV cure-related research at the EOL [3, 17–20].

In this study, we endeavored to understand how various stakeholders perceived HIV cure-related research at the EOL. We also wished to identify ethical and practical considerations relevant to implementing HIV cure-related research at the EOL. To that end, we conducted in-depth qualitative key informant interviews and focus groups among four types of key stakeholders in the United States: 1) PWH, 2) biomedical HIV cure researchers, 3) HIV clinicians, and 4) bioethicists. Each group was queried as to ethical considerations, safeguards, and protections for conducting HIV cure-related research at the EOL to ensure that this research remains acceptable.

## Methods

### Study setting and participants

Using a purposive, non-probabilistic sampling technique, we conducted 20 key informant interviews from the four groups described above and 3 virtual focus groups with PWH. We recruited participants in a purposive way because most had prior exposure to the topic of HIV cure-related research at the EOL; thus, this research was not a foreign concept to them. We used this purposive sampling technique to obtain triangulated viewpoints because little was known about the ethics and perceptions of conducting HIV cure-related research at the EOL. We selected participants from diverse groups, including academic institutions, HIV clinics, funding agencies, community-based organizations, and community advisory boards across the United States.

We employed a qualitative approach because of the study’s formative nature and the dearth of a priori data relevant to the ethics of HIV cure-related research at the EOL [21]. Empirical ethics places normative ethics, i.e., how one should morally act, within the context of the “real world” and evaluates what people think ought to ethically occur [22]. Key informant interviews and focus groups produced in-depth opinions from a vast array of informants and stakeholders. Further, eliciting empirical research ethics considerations for such a novel topic lends itself to qualitative inquiry because the nuances of such considerations could only be captured through rich input from participants [23].

### Participant recruitment

#### Key informant interviews

The study’s principal investigator (K.D.), in collaboration with a Scientific Advisory Board and community co-investigators (J.T., C.C., and A.K.), identified and sent email invitations to potential key informants to participate in this empirical research ethics study. Upon acceptance of the invitation, we scheduled interviews with participants and provided them with a copy of the institutional review board (IRB)-approved informed consent form, demographic questionnaire, and interview guide.

#### Virtual focus groups

Since 2017, our study team collaborated with two community groups based in Southern California who have been actively advising on the Last Gift study. These included the AntiViral Research Center (AVRC) in San Diego, CA, and the HIV + Aging Research Project – Palm Springs (HARP-PS). Both community groups assisted in the design of the present study, including reviewing the proposed guide (Table 1). In addition, the leaders and coordinators of each respective community group assisted with the logistical arrangements of the virtual focus groups including scheduling, member availability, and completion of informed consent forms and demographic sheets.

**Table 1:** IRB-approved interview guide and focus group question route

Ethical and practical considerations for HIV cure-related research at the end-of-life
**Introduction**
Can you please describe your involvement in HIV (cure)-related research?
Are you familiar with the type of research discussed above? [If yes, move to next question. If no, discuss more]
What, if any, concerns do you have about this sort of research?
Do you think this research should be done [or not]? Why do you think/feel that way? Please explain
**HIV cure-related research at the end of life** (*Ask for explanation after every answer: Why do you think/feel that way?*)
What can be done to ensure these types of studies are implemented effectively?
What can be done to ensure these types of studies are implemented in an ethical way?
What can be done to ensure these studies remain patient/participant-centered?
What can be done to ensure these studies remain socially acceptable?
How should we navigate the potential conflicts between research aims and clinical care needs? Who should decide?
What about advance directives? How do they relate to the priority of the research aims?
What about palliative care? How does it relate to the priority of the research aims?
**Additional considerations** (*Ask for explanation after every answer: Why do you think/feel that way?*)
What should the role of the HIV care provider be in this type of research?
Do you think EOL research could also be relevant to other fields? Why or why not?
Do you think cultural differences play a part in how people view this research? If yes, how so?
How do you think COVID-19 might affect perceptions around rapid research autopsy programs?
What are the ethical issues brought about by medical-assistance-in-dying–now (MAiD) legal in California and Canada?
**Wrap up and closing**
Would you like to add anything or make additional comments?

### Data collection

We conducted and recorded all interviews and focus groups via a Health Insurance Portability and Accountability Act (HIPAA)-compliant virtual conferencing platform in the English language. We used an IRB-approved interview guide to facilitate each interview and focus group. In addition, we used IRB-approved PowerPoint slides to guide the virtual focus group conversations, as these were more community-friendly than a lengthy guide.

Two members of our research team (K.D. and J.K.) conducted each interview and virtual focus group, and guided informants by asking open-ended questions. Interviewers (K.D. and J.K.) kept detailed field notes for each interview and focus group. Community members received compensation in the form of an electronic $20 Visa gift card; informants representing research institutions or funding agencies received no compensation.

### Data Analysis

Following each interview and focus group, we saved the audio file to a secure drive which only two members of the research team (K.D. and J.K.) could access. A member of the research team (J.K.) then uploaded the file to a secure transcription service’s encrypted website for verbatim transcription. One researcher (J.K.) reviewed each transcript for completeness and accuracy by vetting the corresponding audio recording against the transcript. We did not return transcripts to participants for comment or correction and participants did not provide feedback on the findings. Because of the novelty and exploratory nature of this study, we employed conventional thematic analysis involving inductive reasoning as our methodological approach to understand and distill the emergent data [21].

Our team compiled all de-identified answers into one master document for manual coding. We employed a high degree of fidelity during the questioning process, following the guide. This allowed us to organize our master transcript document by collating all responses for each question of the guide. Responses were organized by informant types, allowing us to review the range and richness of responses obtained. After deep refamiliarization with the transcripts, two members of the research team (K.D. and J.K.) double coded the data and organized them into emergent themes and subthemes via an inductive approach. Due to the novelty of the research, we did not use a pre-existing coding scheme. The primary investigator (K.D.) acted as the primary coder, and a research team member (J.K.) acted as the secondary coder. The primary coder derived the key themes and sub-themes that emerged, generated the initial code book, extracted salient quotes, and parsed out key considerations and safeguards for conducting HIV cure-related research at the EOL. The secondary coder reviewed the primary coder’s assessment, made refinements as necessary, and organized quotes into a tabular format. The coding team (K.D. and J.K.) resolved discrepancies by discussion and consensus during bi-weekly virtual meetings to reach validity, reliability, and consistency in the interpretation of the data, until complete agreement was reached. The most illustrative quotes associated with major themes can be found in the results section. Supplementary quotes are included in the Additional file 1: Table S1**.**

### Ethics statement

This study was performed in according with all relevant guidelines and regulations, such as the U.S. Code of Federal Regulations and the Declaration of Helsinki. The University of North Carolina at Chapel Hill (UNC-CH) IRB approved this empirical research ethics study (study #19-0522). All key informant interview participants provided verbal consent. Verbal consent was IRB-approved and recorded on the participant’s audio file. All virtual focus group participants provided written consent, and additional data security procedures were sent to them prior to each virtual focus group. All participants received a copy of the informed consent form prior to their interview or focus group. To protect the confidentiality of all study participants, we de-identified all study-related documents and transcripts, and destroyed all audio files once transcription was completed and checked for quality.

## Results

Interview participants included 14 cisgender men and 6 cisgender women, most of whom were Caucasian/non-Hispanic (Table 2). We recruited 14 biomedical HIV cure researchers, 4 HIV clinicians, 1 community member and 1 bioethicist. Interview participants worked in the field of HIV for a mean of 22 years (SD: 10.1 years), and in the field of HIV cure-related research for a mean of 8.8 years (SD: 7.9 years). Virtual focus group participants (all community members) included 11 cisgender men and 5 cisgender women with HIV aged 47–78 years (Table 3). Of these, 10 were Caucasian/White, 5 were African-American/Black, and 1 was American Indian/Alaska Native and Hispanic/Latino descent.

**Table 2:** Demographic characteristics of key informant interview participants (United States, 2020)

Participant number	Sex	Race/ethnicity	Informant type
101	Male	Caucasian/non-Hispanic	Researcher
102	Male	Caucasian/non-Hispanic	Bioethicist
103	Female	Caucasian/non-Hispanic	Researcher
104	Male	Caucasian/non-Hispanic	Community member
105	Male	Caucasian/non-Hispanic	Researcher
106	Male	Caucasian/non-Hispanic	Researcher
107	Male	Caucasian/non-Hispanic	Researcher
108	Female	American Indian/Hispanic	Researcher
109	Male	Caucasian/non-Hispanic	Researcher
110	Male	Caucasian/non-Hispanic	Researcher
111	Female	Asian	HIV clinician
112	Male	Caucasian/non-Hispanic	Researcher
113	Male	Caucasian/non-Hispanic	Researcher
114	Male	Caucasian/Hispanic	HIV clinician
115	Female	Caucasian/non-Hispanic	HIV clinician
116	Male	Caucasian/non-Hispanic	Researcher
117	Male	Caucasian/non-Hispanic	Researcher
118	Male	Caucasian/non-Hispanic	Researcher
119	Female	Black/African-American	Researcher
120	Female	Caucasian/non-Hispanic	HIV clinician

**Table 3:** Demographic characteristics of focus group participants (Southern California, 2020)

	FG-1	FG-2	FG-3	Total	Percent
*n*	6	3	7	16	
Gender					
Male	4	2	5	11	68.8
Female	2	1	2	5	31.3
Transgender (male to female)	0	0	0	0	0
Transgender (female to male)	0	0	0	0	0
Gender queer/non-binary	0	0	0	0	0
Did not specify	0	0	0	0	0
Age (median: 58; range: 47–78)					
40–49	0	1	1	2	12.5
50–59	2	1	2	5	31.3
60–69	1	1	2	4	25.0
70–79	0	0	1	1	6.3
Did not specify	3	0	1	4	25.0
Ethnicity^a^					
Caucasian/White	5	2	3	10	62.5
Black/African-American	0	1	4	5	31.3
Hispanic/Latino Descent	0	0	1	1	6.3
American Indian/Alaska Native	0	0	1	1	6.3
Native Hawaiian/ Other Pacific Islander	0	0	0	0	0
Asian/Asian Descent	0	0	0	0	0
Other	0	0	0	0	0
Did not specify	1	0	0	1	6.3

^a^Some participants identified with more than one group

### Perceptions of HIV Cure-related research at the EOL

#### Whether HIV cure-related research at the EOL should be done

All participants were generally supportive of conducting HIV cure-related research at the EOL. A focus group participant described this research as a “unique and groundbreaking way of doing research” (FG-1 participant). Likewise, all clinicians and biomedical HIV cure researchers in our study echoed this favorable perception:

*[One] of the reasons that HIV clinical care has advanced so much to the point where it's no longer a death sentence and benefited the lives of so many millions of people is that, as a community, people that live with HIV have given of themselves to participate in HIV-related studies. That has provided so much knowledge, information about the disease. I think, in the same manner, individuals that are end-of-life would probably, in the same vein, be altruistic in the same way that people that live with HIV historically have been. About giving of themselves.* – HIV clinician #114

*[S]ome people with HIV do express interest in this… feel that it's a way to give back to the community. The HIV population is very activist, a scientific activism, and have been unusually involved and interested in research design. So, I think it is a very reasonable thing to do to try to accommodate those interests.* – Researcher #101

Altruism has long been a major motivating factor for PWH to engage in research [24]. A researcher (#16) noted that EOL HIV cure research may simply represent “a natural progression” for PWH. Most researchers, HIV clinicians, and the bioethicist recognized the necessity of genuine altruism involved in HIV-related research:

*You have to be an altruistic person to participate in research just to begin with. And I think taking advantage of altruism for a good outcome and a good cause is not really taking advantage of it. I think it's leveraging altruism.* – HIV clinician #115

An HIV clinician suggested that researchers acknowledge their participants’ altruism and contributions to HIV research. This altruism has led to today’s situation of extremely effective antiretroviral treatment:

*Especially, looking at the HIV epidemic, we've gone from people who are stigmatized, marginalized, to being extremely important members of the scientific community. That is something that they can feel very proud of. It's something that they can recognize as part of their accomplishments in this lifetime. While we don't want to take advantage of people, period, to acknowledge that altruism, I think, is completely fine. I think that that actually may even be a plus.* – HIV clinician #120

Some biomedical HIV cure researchers noted the parallel between HIV cure-related research conducted at the EOL and cancer research conducted at the EOL:

*Well, I see a parallel with cancer research. And so, yes, I think you're going to find a lot of folks living with end stage HIV or complications at the end-of-life chronic conditions or acute conditions that they will want to participate also even knowing that it could cut some of their remaining life short. *– Researcher #108

Overall, representatives from all four key stakeholder groups reacted positively to HIV cure-related research being conducted at the EOL. Their support was based on the historic altruism of PWH and the significant knowledge to be gained by conducting theses studies. Associations were made between this type of research and cancer research conducted at the EOL. Although participants generally supported this research, their support was not free from concerns.

#### Concerns about HIV cure-related research at the EOL

All four stakeholder groups expressed concerns about HIV cure-related research at the EOL, but each group’s concerns diverged from the other groups. A point of convergence, however, was the need for robust community engagement.

*So I strongly believe in the value of engaging the community in the formative stages of research, particularly when it relates to potentially controversial issues.* – HIV clinician #111

Limited public knowledge of HIV cure-related research concerned one researcher:

*I'm also cautious about what would be the perception of this in the public, the funders, all the stakeholders that are supporting [this] research in general.* – Researcher #110

Undue influence was a potential worry expressed by the bioethicist (#102) and some HIV clinicians, particularly if the “discontinuation of [ART]… or a harmful treatment is being proposed” (HIV clinician #111). The bioethicist (#102) added that “there is a layer of societal expectation” regarding HIV that may contribute to “circumstances… where you could feel an added obligation to participate in a research study.”

One community member with HIV, however, cautioned researchers and regulators about being overly paternalistic around EOL HIV cure research:

*[M]y concerns are not about vulnerability so much as they are about allowing people to have agency and make their decisions… to be able to participate. Because at this stage in life, they really don't have a lot to lose, and they have so much to gain by participating.* – Community member #104

Likewise, the adequacy of the informed consent was of concern to researchers and HIV clinicians.

*Consent is paramount [and participants must be] fully aware of the study, the study protocols, the meaning of the study, what it means for the study itself, [and] what it means for them.* – HIV clinician, #114

Representatives of all four key stakeholder groups expressed concerns about conducting HIV cure-related research at the EOL. Though there was little convergence on this topic, participants clearly recognized the importance of early community and stakeholder participation and involvement. These concerns qualified their general support for this research and did not negate it.

### Considerations for HIV cure-related research at the EOL

#### Effective Implementation of HIV cure-related research at the EOL

All participants provided considerations about how to effectively implement HIV cure-related research at the EOL. The need for community and stakeholder involvement was reported by all categories of informants.

*But honestly, I think community involvement is probably what I would say is the most important because … they [community members] put boundaries on what we want to do and what we should do.* – Researcher #119

Transparency and open communication were perceived as a part of community and stakeholder involvement. To illustrate the importance of communication, one researcher described their involvement with the Last Gift study:

*[I]t's that very important communication. A lot of it is one-on-one, phone calls, we send birthday cards, we speak to the next of kin if they have someone living in the home too, we communicate with their care providers when given permission to do so, we communicate with their next of kin when given permission to do so.* – Researcher #108

In addition to community involvement and clear communication, most clinicians and researchers mentioned the diversity of participants as being integral to the success of EOL research. One HIV clinician also posited redefining EOL research to encompass the geriatric population in addition to those with a terminal illness:

*So, I think one of the things that I've noticed for a lot of the end-of-life research and it makes sense, is often we're almost always engaging someone who knows that the end of life is coming, they've been handed a big diagnosis of some kind. And they've months, maybe years left to live. I do wonder if some of these things can be brought in earlier when someone doesn't have a, "My life is ending soon but I'm getting into the twilight years of my life." As a result, are we focused on a population where life looks a little bit different anyway? People who know that they have some sort of diagnosis that's terminal or whatever it is.* – HIV clinician #115

One researched (#116) mentioned the need for multi-disciplinary research teams as important to ensure effective implementation of EOL HIV cure research. Further, one HIV clinician expressed the desire for closer and more open relationships with researchers:

*[B]ecause we're people who have built, sometimes, many, many years-long relationships with patients, and having our involvement, and having us be on board to refer to a study, I think, is really essential*. – HIV clinician #120.

Informants recommended strong stakeholder and community involvement with transparent communication between the research teams and the community as two critical components to ensure effective implementation of HIV cure-related research at the EOL. Respondents also recognized the need for diversity of study participants and multi-disciplinary research teams as necessarily important, as well as establishing strong relationships between the research teams and HIV clinicians.

#### Ethical Implementation of HIV cure-related research at the EOL

When asked what could be done to ensure ethical implementation of HIV cure-related research at the EOL, most researchers acknowledged the sensitivity of the topic and stated that “we have to be very careful in [our] approach[es]” (Researcher #110). The bioethicist echoed this sentiment:

*I think that the challenge is how to do it, not whether it should or should not be done. I think that it's useful research and we should try and find a way to do it.* – Bioethicist #102

Of note, one researcher (#112) perceived an ethical responsibility of researchers to seek an HIV cure particularly for low and middle income countries (LMIC) where the need, or social value, may be greatest.

Other ways to ensure ethical implementation of EOL HIV cure research varied, but many dealt with the study protocol. The bioethicist (#102) pointed out that substantial knowledge must be learned from the research, and findings must lead to useful and actionable results:

*So, if you did this study and learned nothing, or you learned something that could never be applied, and that was all you could ever learn from it. That would be a study that would be hard to justify.* – Bioethicist #102

Most HIV clinicians and researchers also mentioned increasing the societal benefits and decreasing the associated clinical risks for participants:

*Before enrolling participants, all of the possibilities would need to be addressed, all of the risks and all the benefits and all the alternatives would need to be carefully addressed. [I]t's important that the strategies are well-vetted and that any interventions are evidence-based and that they have sufficient data behind them, so as not to cause excessive morbidity or excessive damage, discomfort, [or] pain to the participant.* – HIV clinician #114

Each of the key stakeholder groups, except for community members, stated that the risks of undue influence and therapeutic misconception had to be minimized to ensure ethical implementation:

*You don't want someone to feel pressure to participate in this research. You don't want somebody to be participating under misconceptions.* – HIV clinician #120

Most community members, HIV clinicians and researchers frequently voiced having robust ethics steering committees, IRBs, and/or data and safety monitoring boards (DSMBs) that include bioethicists, community members, and PWH to oversee the research and ensure ethicality at the EOL:

*I think having a study steering committee made up of people that have an ethics background, as well as community members, and other involved persons. So maybe family members of the persons that are being recruited, who will serve either as part of the DSMB, or as separate advisory committee throughout the study, is one way to ensure that the study is being performed ethically. *– HIV clinician #111

Several focus group participants noted that aging PWH should not be automatically considered a vulnerable population:

*I think people with HIV are uniquely less vulnerable because they've been, they've faced death before. When they got diagnosed… it was considered as a death sentence and many of them spent, you know, many years helping friends and loved ones die. So, they know what it’s like… So, I think the participants themselves are less vulnerable [than] the family members (laughs).* – FG-1 participant

Two researchers echoed the view that PWH at the EOL were not inherently vulnerable.

Informants recognized the sensitivity of HIV cure-related research at the EOL. To ensure its ethical implementation, researchers must ensure that substantial knowledge be gained from their studies, maximize benefits while minimizing risks, seek to minimize the risk of therapeutic misconception and undue influence, and develop robust steering committees to oversee research protocols.

#### Ensuring HIV cure-related research at the EOL is attuned to the needs of study participants

Open dialogue and understanding emerged as a point of convergence among all stakeholder groups to ensure EOL HIV cure research remains attuned to the needs of study participants. Participants considered open dialogue as necessary not just between the researchers and the patient/participant, but also between the researchers, family/loved ones of participants, and the community as a whole. In particular, HIV clinicians encouraged researchers to be empathetic and to provide periodic updates throughout the study:

*But your research coordinator and your outreach people have to be just people with hearts of gold. And so, to me the study team matters quite a bit to make sure that it remains patient-centered. *– HIV clinician #115

Some focus group participants viewed adaptable and flexible protocols as necessary for conducting HIV cure-related research at the EOL to ensure the comfort of the patient/participant whose health is in decline. They also recommended that participants should have the right to withdraw from the study at any time.

Lastly, some sort of acknowledgement and recognition of PWH who participated in HIV cure-related research at the EOL was resoundingly stated by community members to ensure studies remain centered on the participants.

Overall, informants described establishing an open dialogue and understanding between research teams, their participants, and the community as foremost considerations. Researchers should also be empathetic towards their participants and flexible with their protocols to ensure their participants’ comfort. PWH should also be recognized for their participation in HIV cure-related research at the EOL.

#### Ensuring social acceptability of HIV cure-related research at the EOL

According to all four key stakeholder groups interviewed, transparent communication with the public on issues surrounding HIV, science, and medicine was the way to ensure social acceptability of HIV cure-related research at the EOL:

*We need to, as frequently as possible, engage the general public about science in general, about the status of HIV, science, and medicine. Where we are and what we don't know, and about the opportunities we have and what we can learn. So that they are aware of the risks, the costs, the benefits associated with where we are and where we might be with this disease based on this research. *– Bioethicist #102

An HIV clinician (#115) suggested that researchers be cognizant of the fact that research with participants who are at/near the EOL is an emotionally-charged topic when determining how to address the public. One researcher suggested using personalized stories when communicating about EOL HIV cure research:

*Well, I think that you lead with people's stories. You don't introduce the topic as we decide to do experiments on people at the end of their life. You start with stories… They describe the stories of people. First, they have a great want to give something back at the end of life. There's a lot of gratification that people get from that, a lot of meaning. I think that leading with those stories is always the most important thing to do first. Then you can talk about the science and what that contribution that they've made has been able to do. But introduce the story and make it about them because it is about them. *– Researcher #113

Communication in a decidedly transparent and honest manner with the public was a point of convergence to ensure social acceptability among all respondents. Stakeholders suggested clearly communicating in lay terms how the participants’ generous gift helped advance the search towards an HIV cure.

#### Navigating potential conflicts between research aims and clinical care needs

We asked stakeholders how best to navigate conflicts that may arise between the research aims and clinical care needs with PWH at the EOL. Participants across all categories generally agreed that the patient/participant should decide whether to continue with the research should conflict arise between research aims and clinical needs of the patient/participant.

In particular, the bioethicist (#102) recommended dealing with situations on a case-by-case basis. One clinician (#115) recommended strong communication between the research teams, HIV clinicians, and participants when navigating a conflicts and decisions. One researcher (#108) noted that ethics review boards/IRBs are always available to deal with any type of conflict that may arise.

Overall, informants recognized a participant’s autonomy in deciding whether to continue with research, but also valued communication between the research team, the participant, and the participant’s clinical care provider as being key to resolving any of these potential conflicts.

#### Role of advance directives in HIV cure-related research at the EOL

We asked informants about their perceptions of advance directives in HIV cure-related research conducted with PWH at the EOL. Although the bioethicist (#102) noted that “most people still do not have advance directives”, there was overall support for advance directives in general across all categories of participants.

*[C]ertainly advance directives are important. I actually would suggest if they don't have one, when they get involved in this type of research that they get one* – FG-3 participant

The bioethicist, however, cautioned that advance directives may lack specificity for the EOL HIV cure research context:

*And most of the advance directives that do exist, are almost undoubtedly not specific enough to address what we're talking about. So then… we can't put very much weight on that advance directive at all. However, if we had an ideal world in which people as part of forming an advance directive, have deep and rich conversations with their loved ones, with their family, with their friends. And perhaps articulating the very specific things in the advance directive that are needed, then you can put more weight on it.* – Bioethicist #102

Nevertheless, most HIV clinicians and researchers viewed advance directives positively because they serve as a catalyst for meaningful conversations and reduce the potential for conflict. Importantly, most researchers advised that advance directives be drawn separately from the needs of the research. One researcher viewed conversations between the patient/participant and their primary provider as the appropriate forum for initial discussions regarding advance directives:

*I think the first time a patient sees an advanced directive, needs to be from their clinical provider, their primary care person. And just so that it's not novel or associated with research or associated with the study.* – Researcher #105

A clinician (#114) encouraged research teams to inquire about advance directives to ensure they “would be consistent with any participation… in the research study” (HIV clinician #114). A focus group participant (FG-3) suggested researchers be directed to “check back in with [participants to ensure] that their advance directives [are] still up to date and [that] their wishes are still the same.” In any case, most participants viewed advance directives as taking precedence over the aims of the research protocol.

#### Role of palliative care in HIV cure-related research at the EOL

Most clinicians and researchers strongly valued palliative care for its service to people at the EOL. In particular, most clinicians viewed palliative care as complimentary to EOL research:

*Palliative care is an important adjunct to it [research], I guess. I think comfort remains the primary goal. At least to me, I don't think that you're at cross purposes there.* – HIV clinician #120

Further, most clinicians and researchers emphasized good communication with the palliative care team, because participants may experience chronic pain at the EOL:

*If there [are] things that come up and issues that come up, they can be addressed by staff, from both the research study and from the palliative care team. But I don't think that they necessarily have to… exclude the other. They just would need to go hand in hand and be modified as they go.* – HIV clinician #114

Researchers and HIV clinicians recognized the importance of palliative care in ensuring participants’ comfort in HIV cure-related research conducted at the EOL. Not one of the HIV clinicians or researchers interviewed had a negative view of palliative care. Further, they strongly suggested close collaboration between the research and palliative care teams.

### Additional considerations for HIV cure-related research at the EOL

#### Role of HIV care provider in EOL translational research

When asked whether HIV care providers should play a role in EOL translational research, community members, researchers, and HIV clinicians alike responded in the affirmative because of the close relationship that many PWH develop over many years with their HIV care providers:

*We're a very important part of the patient's team. We, in many ways, may be even part of their family, how they define family, for some of us had been working with these people for literally decades.* – HIV clinician #120

Participants diverged, however, in their views on the type of role the HIV care provider should play. One researcher stated that the main role of HIV care providers in EOL translational research should be to recruit participants by providing their patients with information about such studies:

*If I can ask them to do something, is to inform the patient about the possibility of being involved in such studies… I think this is actually the link you want to establish between the medical doctor and the patient... That these types of studies exist, and that they could be involved if they'd like to. I think that the care provider role is really information at this stage.* – Researcher #117

Another researcher suggested that the HIV care provider be a partner to, or part of, the research team:

*I think it would be nice if the care provider were a partner in it, because I think it is hard for the participant to be going through end-of-life research and not have their primary care provider on board. I think this is the exact same question as next of kin… They just need to be part of the team, because I think it's asking a lot of the participant to do this alone.* – Researcher #119

Yet another researcher, however, believed that the HIV care provider’s role is and should remain separate and distinct from that of the research team:

*Well, they should be supporting research, but I am a big proponent of try to keep the provider separated from the researcher because they have two different roles.* – Researcher #103

One community member, while acknowledging that HIV care providers have a role to play, pointed out that they may have differing views from their patients which could potentially lead to conflict:

*I can see situations where somebody has a really close long-term relationship with their provider. So, I think they will be an important source of knowledge and kind of a sounding board for what's being suggested… There's a possibility that the care provider might not have the same views as the participant 'cause they're focused on keeping their patient alive and not putting them at risk and so forth. So I can see a possibility for conflict there as well… So we wanna minimize that as much as possible because that would be distressing for the participant if they have a close and trusting relationship with their primary provider.* – Community member #104

Whatever the extent of the HIV care provider’s role, most HIV clinicians and researchers regarded open communication between them as an integral component of successful EOL research and one that both parties should embrace.

Despite their divergent views on what role the HIV care provider should play in HIV cure research at the EOL, participants expressed broad support for involving the HIV care provider in some way and establishing open lines of communication between the HIV care provider and the research team.

#### Relevance of EOL translational research to other fields

When asked whether they believed EOL translational research could inform other fields, informants answered resoundingly in the affirmative and suggested many other fields in which the EOL translational model may prove relevant, such as other infectious diseases and non-communicable diseases:

*Research at the end of life I think could really revolutionize a whole bunch of fields, not just for infectious diseases, such as hepatitis or malaria or whatever. But it can also revolutionize what we learn about diabetes.* – Researcher #109

*[I]magine cardiac disease. And, when we get into special organ development by regenerative engineering, it could be very valuable. Putting in an artificial kidney, in situ.* – Researcher #118

One researcher also noted the strong sense of community and altruism that exists among PWH and that this may not be present in other fields:

*I do think that HIV has a special place because HIV has such a strong community component and not every field has that. And so maybe not everyone will be willing to sacrifice their time at the end of life for their community if they don't have such a strong connection.* – Researcher #103

Overall, participants expressed hope that EOL translational research may be relevant to other medical fields, such as hepatitis, cardiac disease, diabetes, and rare diseases among others. They also noted that PWH share a strong sense of community that may not be as strong in other fields, and that this may hamper efforts to translate lessons learned from HIV cure-related EOL research to other fields.

#### Cultural considerations in EOL HIV cure-related research

We asked if cultural differences affected the way people view HIV cure-related research at the EOL. Participants reached a point of convergence on this issue and believed that cultural differences absolutely affect participants’ willingness to engage in this research and their communities’ acceptance of this research.

Participants noted differences in individualistic versus collectivist societal views, as well as differences in the way death is viewed, as being important cultural considerations. Religion was also perceived to factor heavily into our discussion of cultural differences’ impact on HIV cure-related research at the EOL.

Participants also considered medical mistrust, particularly among African-Americans, to be one of the largest cultural barriers to be overcome by actively engaging communities early and throughout research efforts.

*In black populations as another example, there is an issue of medical mistrust, that they view that the health system is untrustworthy and validly so because of systemic inequalities in healthcare.* – HIV clinician #114

In sum, participants regarded cultural differences, such as religion and medical mistrust, as heavily influencing participants’ decisions about engaging in HIV cure-related research at the EOL, as well as their communities’ acceptance of the research. Participants suggested that research teams actively engage communities early and throughout research efforts.

#### COVID-19 and rapid research autopsies

We asked participants how COVID-19 might affect perceptions around rapid research autopsy programs. A community member (#104) and a researcher (#119) noted that discerning whether a participant had COVID-19 at the time of death was of paramount importance to ensure the safety of the research team, even though this may delay collection of tissues.

One clinician was concerned by the rampant disbelief in the science related to COVID-19 and its inflammation of medical mistrust:

*Much has been written about the fact that we're almost fighting two struggles on two fronts, is the pandemic itself and then the public disinformation or the public mistrust surrounding it… I think the current pandemic shows that there is a high level of distrust among the general population, I think it's difficult to quantify just how much.* – HIV clinician #114

One clinician (#120) and a focus group participant (FG-2) expressed concern that COVID-19 affected dying and its associated rituals. Two researchers noted that the scientific synergies between COVID-19 and HIV.

*The more we learn about COVID and how it's doing the thing that it's doing, the more we'll learn about HIV.* – Researcher #116.

*Maybe we can even talk about COVID therapy or a vaccine against COVID to be tested at the end of life*. – Researcher #103.

Participants expressed that the COVID-19 pandemic has affected rapid research autopsies by requiring testing for COVID-19 before performing an autopsy to protect the safety of the medical team. The widespread scientific disbelief surrounding COVID-19 concerned some participants, as did its disruption of rituals surrounding death. However, researchers noted the potential scientific synergies between COVID-19 and HIV.

#### Perceptions of medical-assistance-in-dying (MAiD)

Medical-assistance-in-dying (MAiD) is the process by which terminally ill adults request and receive medication to bring about their own death. This process is legal in Canada and the State of California. We asked participants to describe the ethical issues brought up by MAiD within the context of HIV cure-related research at the EOL. Nearly all of our participants across the four categories supported this practice and noted the patient/participant’s autonomy and “a real sense of control” (FG-2 participant) in deciding how and when to end their life.

One researcher qualified their decision to involve the NOK in the decision and another was concerned by potential differences of opinion between the patient, care provider, and research team:

*Well, so totally the patient's decision, of course. I think that the next of kin should definitively be involved in the decision, again with everything, and which will carefully listen to what they have to say… It is, because you open yourself to vulnerability if the family disagrees and you still move forward with the aid in dying, right? But this is why I think we need more than one witness. We need more than one doctor to agree to do it so that all the fault doesn't go on one person.* – Researcher #103

*A lot of perspectives with potentially conflicting interests. And without having thought it through before, I can imagine possibly situations where, for example, a physician providing care, specifically who's tasked with assisting with death, might disagree with perhaps even the patient him or herself, and the researchers about the potential benefit… That would be a particularly difficult situation to deal with. [C]ertainly I think an attempt should be made to get consensus from those three different parties. *– Researcher #101

As for the safeguards that needed to be in place, most HIV clinicians and researchers agreed that the provision of MAiD should be completely separate from the decision to participate in research. Most HIV clinicians and researchers also recognized the potential needs for honest communications with the patient/participant, mental health evaluations, and/or independent review committees with the decision to undergo MAiD.

One researcher reflected on the positive experience they had with a Last Gift participant with amyotrophic lateral sclerosis (ALS) who chose MAiD in the name of compassion and dignity at the EOL:

*[He] made that decision, and called us in advance and said, "Today is the day I'm going to swallow my pills." He was on the Last Gift program… All of his friends were around, and he had a lovely last day. In terms of ethical issues, I didn't think there were any. I thought it went beautifully, and I thought that's exactly how it's supposed to work.* – Researcher #119

Respondents were generally supportive of MAiD and of the patient/participant’s autonomy to control their EOL. They also expressed concerns over the effects on the research outcomes and a great sensitivity to keeping the research team divorced from the MAiD decion-making process. Additional safeguards included honest communication with the patient/participant, mental health evaluations, and independent review committees.

## Discussion

Our qualitative study assessed stakeholder perspectives on HIV cure-related research at the EOL in the United States, including critical safeguards that should be in place to ensure such research is implemented effectively and ethically. The empirical ethical considerations presented in this paper augment our previous normative considerations from our review of the literature [3].

Key findings from our study are as follows:

1. All four key stakeholder groups generally supported HIV cure-related research at the EOL because of the history of altruism within the PWH community and the potential for substantial scientific knowledge to be gained, specifically regarding measurement of latent HIV reservoirs.
2. Strong stakeholder and community involvement, including open dialogue and transparent communication, are integral to the ethical and effective implementation, as well as the social acceptability of this research.
3. PWH approaching the EOL should not inherently be considered a vulnerable class and their autonomy must be respected when choosing to participate in HIV cure-related research at the EOL and/or when choosing MAiD.
4. Greater diversity among study participants, as well as multi-disciplinary research teams that include bioethicists, socio-behavioral scientists, and HIV care providers, is necessitated by HIV cure-related research at the EOL.
5. The sensitive nature of this research warrants robust oversight to ensure a favorable risk/benefit balance and to minimize the possibility of therapeutic misconception or undue influence.
6. Research protocols should remain flexible to accommodate participants’ comfort and needs at the EOL.

Our findings reveal overall support and enthusiasm among key U.S.-based stakeholder groups for conducting HIV cure-related research at the EOL because of the potential significant knowledge to be gained by this research. All informants, however, expressed concerns about this research, such as socio-political views of this research as taboo and increased risk of undue influence among others, but ultimately diverged as to what those concerns were. Nevertheless, strong support for stakeholder and community involvement early and throughout the research studies was common to all, as was the need for open and honest communication between the research teams, HIV care providers, patients/participants, and the community. It is also extremely important to note that the concerns expressed, whatever they may be, served to qualify respondents’ support for HIV cure-related research at the EOL and in no way negated it.

Our results illustrate the desire for greater diversity among trial participants in HIV cure-related research studies at the EOL. This is not surprising since the homogenous makeup of EOL trial participants to date and calls for further research into diversity’s impact on EOL issues in other fields has been noted in prior research [25, 26]. In addition, our findings call for multi-disciplinary research teams to address all components of EOL care and research combined, although HIV cure-related research at the EOL does not directly involve palliative care [25].

End of life research is a necessarily sensitive topic that mandates high ethical standards [27, 28]. Involvement of all stakeholders, including PWH and their communities, in the design of the research protocols and throughout the studies is integral to establishing trusting relationships and fostering ethical and effective implementation of EOL research [26, 29, 30]. Our findings indicate that HIV cure-related research at the EOL is equal in this respect and may even require greater attention be paid to next-of-kin/loved ones/intimate partners/families of PWH who participate in these studies [31]. In addition, our informants converged upon the opinion that open and transparent communication in lay terms among all stakeholders is also necessary.

This study’s results also demonstrate that patient autonomy must be respected in HIV cure-related research at the EOL. Casarett and Karlawish noted in 2000 that, “[c]ontrol also becomes increasingly important for many patients as the near the end of life” [32]. As such, PWH who enroll in HIV cure-related research at the EOL often wish to exercise such control and care providers should temper their often overly protectionist and paternalistic views [33, 34]. Though terminally ill individuals have historically been seen as a vulnerable group of research participants [32, 33, 35], our study indicates that aging PWH, who once faced the prospect of death when HIV was untreatable, may not consider themselves vulnerable when it comes to this type of research.

Many PWH participate in these studies as an act of activism and existentialism [36], and a natural progression from past HIV research participation. In the past, PWH would not be candidates for traditional organ donation; now, however, PWH in the United States can legally donate their organs to other PWH awaiting transplant, and many choose to do so [37]. HIV cure-related research at the EOL is very similar to, and is an extension of, organ donation because both are motivated almost entirely by altruism. PWH may wish to be a part of HIV cure-related research at the EOL to continue the strong tradition of altruism that has defined the HIV community since its genesis. While participants in our study understood that researchers are mandated to maximize the benefits and minimize the risks of this research, as is true of all human research [32, 33, 38], they also recognized that altruism can greatly shift the risk–benefit calculation [26, 29, 30, 33, 35, 38], and help alleviate possible concerns around exploitation, particularly in HIV EOL research.

Despite the foregoing, our findings also demonstrate the need for ethical safeguards, such as minimizing the risks of therapeutic misconception and potential undue influence, both of which have previously been identified in aging and EOL literature [32, 33], as well as robust oversight. In addition, respondents called for researchers to be empathetic in dealing with participants in HIV cure-related research at the EOL and to employ flexible, adaptive protocols to manage participant issues that arise during a study [30].

Advance directives respect the dignity and autonomy of a patient enrolled in EOL research [25, 30]. Our results reveal that advance directives are equally or more important in HIV cure-related research at the EOL compared to other fields of biomedical research [31], should be revisited throughout the research process to ensure participants’ desires have not changed, and take precedence over the research aims of the study. Similarly, all stakeholder groups regarded palliative care as important to, and a necessary corollary to, HIV cure-related research at the EOL.

Further, primary care providers may not share the same concerns as do their patients regarding the EOL [13, 14]; as such, provider involvement in HIV cure-related EOL research can often be perceived as a hindrance by the research teams. HIV care providers may feel protective towards their patients, a phenomenon referred to as “gatekeeping” in palliative care research [39]. Nonetheless, our results showed a convergence around the inclusion of the primary healthcare provider in HIV cure-related research at the EOL. Divergent views, however, were expressed as to the exact role and scope of the care provider in this research, although all respondents regarded open and transparent communication [13] between the research teams and participants’ primary healthcare provider as a minimum requirement that must be met, particularly because of the close relationship between many PWH and their longtime providers.

As has been discussed, altruism is a defining characteristic of the HIV community. This desire to help others is demonstrated in our data through expressions of hope that HIV cure-related research at the EOL could prove relevant to other fields of medicine, such as COVID-19, hepatitis, cardiac disease, diabetes, and rare diseases. The strong sense of community that HIV engenders because of its associated stigma and social perception [31] may not be as strong, however, in other disease fields.

Cultural differences likely play an important role in influencing participation in, and community acceptance of, HIV cure-related research at the EOL. Our results also demonstrate a potential solution to cultural differences in this research: active and early engagement of the relevant and diverse communities around HIV cure-related research at the EOL. The theme of robust community engagement emerged strongly and consistently throughout our interviews. As HIV cure-related research at the EOL expands to other geographic settings within and beyond the United States, it will be important to appreciate context-specific and cultural diversity considerations [40] for conducting such research.

Currently, COVID-19 has affected every aspect of life, including HIV cure-related research at the EOL. There was general exasperation expressed over the widespread disbelief in science and the politicization of COVID-19. Autopsies create aerosolization and rapid research autopsies are currently necessary to HIV cure-related research at the EOL [11]. For safety reasons, research teams are required to test the body for COVID-19, a procedure which can place the timely collection of tissues in jeopardy [11]. COVID-19 may be another research field where EOL research may be warranted to advance science [41–44].

Finally, MAiD offers terminally ill PWH a chance for control, dignity and compassion that can improve their experience at the EOL [10, 11]. Our data show a general support for the use of MAiD as an expression of a patient/participant’s exercise of autonomy. The findings also demonstrate concerns over the process by which MAiD is undertaken [15]. To counter these concerns, robust safeguards should be in place according to participants in our study, such as an independent review of the choice to employ MAiD as well as open and transparent communication between the medical professional prescribing MAiD and the patient/participant. In all circumstances, the research team must be divorced from the MAiD decision-making process to prevent the perception of undue influence [11].

### Limitations

We acknowledge several limitations of this qualitative interview and focus group study. Our sample size was relatively small. After 20 in-depth interviews and 3 virtual focus groups, we may not have reached saturation—the point when no new information or themes are observed in the data [45]. Due to time and funding constraints, we limited our sample to these participants given the richness of our data. Further, our pool of HIV clinicians and researchers was severely limited by the COVID-19 pandemic. A valuable group of informants to include in this study would have been partners of PWH; however, our team has a separate ongoing study focused on perceptions of next-of-kin/loved ones around EOL HIV cure research [18, 20]. Participants expressed overwhelming support for HIV cure-related research at the EOL, possibly because of our purposive sampling technique and our requirement that all informants be involved with, or have knowledge of, this research. As such, we are aware that we must remain open to dissenting opinions. Since our informants were affiliated with institutions or organizations in the United States, the considerations generated by this study were likely skewed toward resource-rich contexts. Participants were also largely representative of an older, Caucasion/non-Hispanic population; more research is needed into the opinions of diverse groups on HIV cure-related research at the EOL across a diversity of geographic and cultural contexts. While participants made analogies between EOL HIV cure research and the cancer field, other diseases could also provide interesting comparisons (e.g., amyotrophic lateral sclerosis).

## Conclusions

We are no longer seeking to prevent HIV-related deaths as was the case thirty years ago; PWH can lead long and productive lives due to current ART. We are now attempting to “cure” HIV, and the inherent ethical considerations require thoughtful inquiry. To maximize this research’s impact and social value, we must wrestle with these ethical questions and determine the best way forward. Our empirical research study sought to identify through in-depth interviews and focus groups ethical and practical considerations for HIV cure-related research at the EOL. The summary of these considerations derived from our qualitative data can be found in Table 4; this list is not comprehensive. As this research gets scaled up, more much inquiry will need to be directed towards understanding context-specific and cultural considerations for implementing EOL HIV cure research in diverse settings.

**Table 4:** Summary of ethical and practical considerations for HIV cure-related research at the EOL

Perceptions of HIV cure-related research at the EOL
**Whether HIV cure-related research at the EOL should be done**
Because of the potential scientific knowledge to be gained, HIV cure-related research at the EOL may be ethically permissible, but adequate safeguards must be in place [3]
PWH near the EOL should not be treated as an inherently vulnerable class and should not be automatically barred from participating in HIV cure-related research
**Concerns about HIV cure-related research at the EOL**
Investigators should be cognizant that PWH at the EOL may feel an obligation to participate in the study and be careful of exerting any undue influence
Regulators should avoid being overly paternalistic with PWH as they may desire to exercise their autonomy and agency at the EOL
***Conducting HIV cure-related research at the EOL***
**Effective Implementation of HIV cure-related research at the EOL**
Research teams should engage relevant stakeholders, including community representatives, in the design of protocols, during the studies, and in the dissemination of findings
Research teams should ensure diverse populations are aware of, and have access to, HIV cure-related research at the EOL to ensure justice and equity
Research teams should be multidisciplinary and should engage PWH before the EOL process
**Ethical Implementation of HIV cure-related research at the EOL**
Robust ethics steering committees, IRBs, and DSBMs should oversee proposed studies and ensure studies remain within acceptable risk–benefit parameters
**Ensuring HIV cure-related research at the EOL is attuned to the needs of study participants**
Research teams should place great import on empathy and communication with the participants, their next-of-kin/loved ones/intimate partners [20], and their communities
Research teams need to consider the diverse and evolving needs of patients/participants at the EOL in the protocol trial design and actively engage PWH in designing these protocols
There should be adequate acknowledgement of the extreme generosity and altruism of PWH who participate in this research [24, 34]
**Ensuring social acceptability of HIV cure-related research at the EOL**
Research teams should be very intentional and transparent in their communications with the general public on issues surrounding HIV, science, medicine, and death
**Navigating potential conflicts between research aims and clinical care**
To preserve autonomy, research teams should err on the side of what patients/participants would want at the EOL
Research teams should deal with potential conflicts between research aims and clinical care needs on a case-by-case basis and with the help of bioethicists
**Role of advance directives in HIV cure-related research at the EOL**
Research teams should ask PWH who desire to participate in HIV cure-related research at the EOL whether they have a recent advance directive in place in a discussion that is separate from the informed consent process
**Role of palliative care in HIV cure-related research at the EOL**
Research teams and palliative care teams should remain in communication with each other to ensure that participants remain comfortable at the EOL
**Additional considerations for HIV cure-related research at the EOL**
**Role of HIV care providers in EOL translational research**
HIV care providers should be involved in the EOL translational research process; however, it should be recognized that researchers and providers have two distinct roles that must be made clear to participants to avoid therapeutic misconception
**Relevance of EOL translational research to other fields**
The EOL translational research model may prove highly relevant to other medical fields, such as COVID-19, hepatitis, rare diseases, cardiac disease, and diabetes, among others. Lessons learned from the field of HIV cure-related research may also be translatable to other fields of research
**Cultural considerations**
Cultural differences should be considered when implementing HIV cure-related research at the EOL; research teams should engage and inform communities early and throughout research efforts
**COVID-19 and rapid research autopsy**
Protecting the rapid research autopsy team, ensuring timely collection of tissue samples, and surmounting the widespread disbelief in science should be considered when conducting HIV cure-related research in the COVID-19 era
**Medical-assistance-in-dying (MAiD)**
Additional ethical considerations and safeguards are warranted should a patient choose MAiD within HIV cure-related studies at the EOL; the MAiD decision should be completely divorced from the research process to prevent any perception of undue influence [11]

## Supplementary Information

**Additional file 1: Table S1.** Additional Quotes—Ethical and Practical Considerations for HIV Cure-Related Research at the EOL (United States, 2021)

## Data Availability

All data generated or analyzed during this study were included in this published article and its supplementary information files.

## Abbreviations

AIDS: Acquired Immune Deficiency Syndrome
ALS: Amyotrophic Lateral Sclerosis
ART: Antiretroviral Therapy
AVRC: AntiViral Research Center
COVID-19: Coronavirus Disease 2019
DSMB: Data Safety Monitoring Board
EOL: End of Life
HARP-PS: HIV + Aging Research Project—Palm Springs
HIV: Human Immunodeficiency Virus
HIPAA: Health Insurance Portability and Accountability Act
IRB: Institutional Review Board
MAiD: Medical Assistance in Dying
NOK: Next of Kin
PWH: People Living With HIV
UCSD: University of Southern California—San Diego
UNC-CH: University of North Carolina—Chapel Hill
