# Supplementary material for: Ethical and practical considerations for HIV cure-related research at the end-of-life: a qualitative interview and focus group study in the United States
Source: BMC Med Ethics. 2022 Jan 11;23:2. doi: 10.1186/s12910-022-00741-8 (PMC8748179; doi:10.1186/s12910-022-00741-8)
Supplement: Supplementary file 1 — Additional file 1: Table S1. Additional Quotes—Ethical and Practical Considerations for HIV Cure-Related Research at the EOL (United States, 2021) [file 12910_2022_741_MOESM1_ESM.docx]

**Supplementary Table 1: Additional Quotes – Ethical and Practical Considerations for HIV Cure-Related Research at the EOL (United States, 2021)**

| **Themes and**  **Sub-Themes** | **Participant Number** | **Informant Type** | **Quotations** |
| --- | --- | --- | --- |
| ***Perceptions of HIV Cure-Related Research at the EOL*** | | | |
| **Whether HIV Cure-Related Research at the EOL Should Be Done** | | | |
| Support for HIV cure-related research at the EOL | 102 | Bioethicist | *Yeah. I think it's an opportunity to learn things about the disease that we don't yet understand.* |
|  | FG-1 | Participant | *[W]e can't biopsy while people are alive. So it's just been amazing. I think it continues to be amazing.* |
| Leveraging Altruism | 102 | Bioethicist | *We were always hoping for them to be altruistic. It's probably a little bit of semantics but I mean, we need to take advantage of altruism for any research.* |
|  | 119 | Researcher | *Altruism is a great thing. I think, again, when you put the options in front of someone and explain the true risks and true benefits, the participants that I have talked to at least have impressed upon me that this study was one of the most important things at their end of life.* |
| Parallel with cancer research at the EOL | 112 | Researcher | *It's equivalent to in cancer therapy using late stage metastatic disease patients for new … therapies. We haven't had that in HIV for a very, very long time since the beginning of the disease. And so it sets a very high bar for new therapies to be tested in humans.* |
|  | 117 | Researcher | *I think the cancer field, obviously I would say progressed quite rapidly… The difference here is that for these cancer patients… there is still a potential benefit… [T]hat's a different situation than trying something in people living with HIV who are going to die anyway from something else, and you know that what you're going to give them, which is to target the HIV reservoir is not going to solve their main health issue.* |
| **Concerns about HIV Cure-Related Research at the EOL** | | | |
| General Concerns | 102 | Bioethicist | *So, I mean, a number of your questions asked about what the concerns are. And I [am] actually struggling with this in part because every situation has its unique issues. But if I was looking for general issues, the biggest one for me is that people who are in an extraordinary circumstance, one is a disease that is notorious.* |
|  | 109 | Researcher | *I have concerns about HIV cure related research in general, in terms of safety and efficacy and giving people hope for things that the research really isn't designed to do. In the Last Gift in particular, it sort of overcomes a lot of those issues in terms of participants are well informed about what the risks and the benefits are and that it really on the intervention side, they seem to be more concerned about giving back versus what they can get for themselves, which makes me a lot less concerned around interventions in that setting.* |
| Ensuring robust community engagement | 111 | HIV clinician | *I think where I have some concerns is if the community is not involved in designing the study. So I think as long as the community is engaged in the design of the study, and they help to guide that, I feel much more comfortable with that approach.* |
|  | 108 | Researcher | *The concerns that I have, have to do more with how we communicate with others in the community* |
| Minimizing chance of undue influence | 102 | Bioethicist | *And second for anyone suffering from a terminal illness and how that affects them and their family. What all that means is that there is a risk of people being asked to do things that... So that coercion aspect is what worries me most, even though nobody is coming and saying, “You have to do this.”* |
| Curbing Paternalism | 104 | Community member | *I think most of the obstacles I've seen have been kind of being overly paternalistic from the, the regulators point of view, from the IRBs.* |
| Ensuring adequacy of informed consent | 111 | HIV clinician | *And that’s a little bit of who is hired to do the consenting, and how the training as far as study consent goes, I think that is a really critical part.* |
|  | 115 | HIV clinician | *I am a huge proponent for research. Especially, and I really think end-of-life research is really important. It just has to be done in a way where there is a lot of upfront understanding, mostly with the patient, but probably, to some degree, the provider as well.* |
|  | 103 | Researcher | *I think that the main concern that I will have is to perform proper consent and to make sure to only involve people that are able to provide informed consent …* |
| Minimizing negative effects on patients | 105 | Researcher | *As long as it's consented to and ethically appropriate. The risk-benefit ratio obviously has to be considered.* |
| Minimizing chance of undue influence | 102 | Bioethicist | *And second for anyone suffering from a terminal illness and how that affects them and their family. What all that means is that there is a risk of people being asked to do things that... So that coercion aspect is what worries me most, even though nobody is coming and saying, “You have to do this.”* |
| ***Considerations for HIV Cure-Related Research at the EOL*** | | | |
| **Effective Implementation of HIV Cure-Related Research at the EOL** | | | |
| Ensuring community/stakeholder involvement | 102 | Bioethicist | *And so that would be this is a situation where you might really want to ask that question, should these other individuals have a say in this as well? To what extent should they have that right? And that's really tricky in a country. And this is one of the social contexts. We live in a country that is very individualistic.* |
|  | 104 | Community member | *I think what they've been doing in terms of engaging the community, bringing together all the stakeholders. I mean, they bring together the researchers, the study staff, um, as well as community and, and really make sure that it happens smoothly and that everybody's needs.* |
|  | FG-3 | Participant | *If we're talking about effectiveness, it's really important that, not only does the word get out but it gets out to all communities affected by HIV. So, um, I understand targeting an older population because they tend to be, you know, closer to the end-of-life. But, um, it can't just be one ethnic group or, or one race of people that is invited into the study.* |
|  | 114 | HIV clinician | *It'll be important for participants to have a ready access to study staff, so that if there is any issues at all, any concerns, any anxieties that they can be readily addressed. That's the participant standpoint, I guess, from the standpoint of relieving anxieties.* |
|  | 110 | Researcher | *I think that clearly we cannot do this kind of research without a very strong support of our community or a community advisory board that will help us approach this question and a board that has strong opinion, not yes men. People that are going to raise questions and raise questions not only about HIV, people living with HIV in general because we cannot do a generalization.* |
|  | 119 | Researcher | *I think community involvement, which I think we've done very well. I think getting investigators who are dedicated to ethical conduct of research and not just the ability to obtain rapid autopsies and associated samples.* |
| Ensuring diversity of participants | 111 | HIV clinician | *I think a lot about study recruitment. When you're talking about implementation you can't implement study unless you have people involved. One of the things that we've been noticing, in clinical trials in general, is a lack of... we're having more difficulty in engaging clinical providers in referring their patients to studies. Certainly, you know, it seems that when a provider refers a patient they are more likely to participate, because they trust their own doctor, and they trust that their doctor would not be recommending something that would potentially be harmful to them* |
|  | 115 | HIV clinician | *So, to me, the effective is that, or part of the effective is making sure everyone has access to it and maybe not just people who are truly at the end of their lives. I mean, I think as with anything, we want to make sure all of our populations had access to it.* |
|  | 110 | Researcher | *It's clear it's an important topic that has the potential to advance a lot of the questions that are still pending regarding the reservoir, but … I think that from an ethical point of view, … we have to be very careful in how we move forward with this type of research.* |
| **Ethical Implementation of HIV Cure-Related Research at the EOL** | | | |
| Recognizing the sensitivity of HIV cure-related research at the EOL | 102 | Bioethicist | *My guess is that that number is not going to be huge. It's going to be a fairly small number of people. So that raises a problem for this research, because now it decreases the probability that we'll be able to say something definitive at the end of our study.* |
|  | 110 | Researcher | *This is the kind of thing that we have to deal with and I think we have to be very careful because this type of research has a lot of potential but it takes only one thing that goes bad and everything will bring us back to zero, to the beginning, or even prevent us to do this.* |
| Ensuring substantial knowledge will be gained by the research | 110 | Researcher | *It's clear it's an important topic that has the potential to advance a lot of the questions that are still pending regarding the reservoir, but … I think that from an ethical point of view, … we have to be very careful in how we move forward with this type of research.* |
| Maximizing benefits while minimizing risks | 114 | HIV clinician | *Definitely, there needs to be a full assessment of the risks and the benefits.* |
|  | 101 | Researcher | *I think the main thing is what I've already talked about, which is this consideration of whether there is a likely benefit to society or to the HIV-infected population. I think that should be the principal consideration.* |
|  | 105 | Researcher | *So obviously, again, we have to bring only the best treatments, the things that we believe in the most to participants.* |
|  | 102 | Bioethicist | *And they assume that that doctor is there entirely for their benefit. So if the doctor says, "Oh, here's a research study that you might be interested in," they assume it's in their interest to participate in that research study. And that's part of the so-called therapeutic misconception. So I can see that happening here as well. And what we usually tell people for the case I gave would be, that that's why any question about participating in research studies should come from somebody else, not from their doctor.* |
| Minimizing potential for therapeutic misconception/undue influence | 104 | Community member | *I think again involving the patients and the next of kin as you've been doing. And also engaging the larger kind of community as well in terms of making sure that they buy into it, that they understand it* |
|  | 107 | Researcher | *I think in general, there would have to be some measures of how the study was proposed to this individual so that they didn't feel pressured to do it.* |
| Developing robust steering committees to oversee research studies | 114 | HIV clinician | *I think [having] the standard research protocols of Data Monitoring and Safety Board and IRB and all that, all these things of course are in place to safeguard against unethical research practices.* |
|  | 115 | HIV clinician | *I think this is where IRB oversight is extremely important. But I think probably before it gets to an IRB, and … having huge community engagement before it gets to that place.* |
|  | 115 | HIV clinician | *Make sure the product that goes into the IRB is really vetted by patients living with HIV, some lived experience.* |
|  | 108 | Researcher | *I think it's really important that we have an advisory board and we've been very fortunate to have such a community advisory board of individuals who are currently living with HIV and advanced chronic conditions.* |
|  | 108 | Researcher | *We should be asking a whole bunch from one of those participants because to get the most scientific usefulness out of it, then we're going to need all those assessments. That means that the person really needs to be engaged in the research, understand what it's giving and what it's not giving, then what their risks for the contributions would be. That requires a whole lot on the front end for informed consent and discussions.* |
|  | 110 | Researcher | *I think it's important that the boards that are reviewing those requests and those proposal be well equipped with people that understand what this type research is all about and that have the thoughts and the depth to really analyze what are the pro and the cons and the potential ethical implication that this might represent.* |
|  | 110 | Researcher | *I think it's important that the boards that are reviewing those requests and those proposal be well equipped with people that understand what this type research is all about and that have the thoughts and the depth to really analyze what are the pro and the cons and the potential ethical implication that this might represent.* |
|  | 116 | Researcher | *Well, I think the routine scientific review and then Institutional Review Board review is still the bedrock of these kinds of protocols.* |
|  | 119 | Researcher | *I think getting ethicists involved, not that we can't do ethical science without an ethicist, but I think getting more qualitative science delving deeper into the questions, getting more understanding about the motivations about why people are doing what they're doing and getting a better understanding about whether people truly feel that their wishes are being honored at the end of life. We tend to ask pretty quantitative questions, yes, no, and I think really getting a much deeper understanding from participants, from their loved ones, and to something like this, to some extent the scientists about the whys give us a much better understanding about the research and how it's being done ethically.* |
| PHW are not inherently vulnerable | 105 | Researcher | *[T]here are people much more eloquent than me saying, we've got to stop saying some of these participants are vulnerable. I think that we can safely, ethically enroll participants in these sorts of studies with appropriate informed consent.* |
|  | 107 | Researcher | *I think we've learned with [the] Last Gift [study] that they want a purpose. That's kind of a theme that we've realized throughout this project is that people at the end of life, rather than being this like broken helpless population, are really looking for a purpose at the end of life.* |
| **Ensuring HIV Cure-Related Research at the EOL in Attuned to the Needs of Study Participants** | | | |
| Establishing open dialogue between research teams, participants, and the community | FG-1 | Participant | *Well, here's a website where the information gets posted, or something like that, would, would help kind of close that loop and let people know it's their loved one, you know, contributed towards.* |
|  | FG-1 | Participant | *Certainly, it would be great to respond personally to a family member or family members of a specific participant with respect to what [samples] came from them.* |
|  | FG-1 | Participant | *Maybe a little video once a year or something, just kinda giving in lay language, a status of what's going on with the trials. Or the study and what what it’s meant to people as well as the benefit that it's having to the field. I think that would be really interesting for people to be able to see. 'Cause unfortunately, this is... this really gets into a lot more than just hard science, you know? This is a pretty emotional study for everyone involved.* |
|  | FG-2 | Participant | *And then as like, um, one test is given, um, looking to see if there is some kind of cytokine cloud developing, that, um, just that communication to them immediately.* |
|  | FG-2 | Participant | *It goes back to just that open line of communication with the patient and the family. A lot of emotions are going on during that time, and I think it's important that you will be able to solidify agreement of participating in the program.* |
|  | FG-3 | Participant | *If you want to talk about patient-centered, it has to be, the patient has to be kept informed. So in this case, once they sign up, I think it's important to reach out to them every so often, even if there's nothing really going on or nothing exciting happening, so to speak. That contact is made with them so that they don't feel like they're just kind of adrift in this process and, and, you know, waiting for the end.* |
|  | 114 | HIV clinician | *[S]o that study staff are aware of, not just the participant but the participant in the context of their family and the context of their community …* |
|  | 105 | Researcher | *I think other research that has gone on in the community of people with HIV really shows how important and how useful groups like community advisory boards can be, how important it is to have the community at our scientific meetings. And to some extent, as long as we keep those lines of communication open between the research side and the community, the community is going to tell us when we're crossing the line or not going up to the line close enough.* |
|  | 108 | Researcher | *And so, I hear very directly what their concerns are of the case managers and the providers as well as the actual individuals who are living with this chronic condition. So I think keeping that open dialogue.* |
|  | 109 | Researcher | *Again, I think involving them in both the study, but I think more importantly in the design of the studies. So, not just along the way, but are we addressing questions that are important to them, not just questions that are important to us. How could end of life research benefit the participant? Why is it important to them? How would they like this kind of study to be structured?* |
|  | 110 | Researcher | *We have to make sure that this is done in such a way that the person is really at the center and what they want is really respected.* |
|  | 119 | Researcher | *I think that stays patient-centered, keeping them involved in the structure of the study, the inclusion, the exclusion criteria. Again, that allows us, in my opinion, to find that there is equipoise within the community for the kinds of questions that we're asking and the way we're structuring the study.* |
| Ensuring that researchers are empathetic towards their participants | 104 | Community member | *Um, she's really the kind of the heart and soul of, um, the whole study, especially from the patient perspective. 'Cause she's the one that in- interacts with them. So ensuring that you have someone of that caliber and being able to duplicate that person (laughs) in case, you know, anything happens to them or if you want to expand the study to make sure that you have, um, you know, a set of, of, you know, job requirements* |
|  | FG-2 | Participant | *I think from an emotional point of view supporting and encouraging them through that process. Because if they're gonna be experiencing the discomfort from that type of treatment in addition to the, the symptoms and things they're already dying from ….* |
|  | 111 | HIV clinician | *[C]ommunicating what's going on and results in a way that the community can understand exactly what's happening, I think are all ways to ensure that it remains ethical throughout the study and remains patient centered.* |
| Ensuring protocols that are adaptable and flexible | FG-2 | Participant | *And whatever you can do to make them more comfortable and letting them know upfront what those options are. You know, that they can ask for it, that sort of thing. So, they know they have control over the process.* |
| Ensuring participants can withdraw from the study at any time | FG-2 | Participant | *And of course, they can stop at any time, right? So, if it's too much, they just say, "I don't want to do this anymore," and we respect their wishes.* |
| Ensuring the comfort of study participants | FG-2 | Participant | *You just employ the same palliative care that you give to anybody end of life. And very aggressively, you know, treat pain. Don't be afraid to use opioids, things like that. Um, so that the primary consideration is keeping people comfortable …* |
|  | 107 | Researcher | *So I think kind of those two things are important just how to protect them even though we know that there's less long-term issues because of their shortened lifespan…* |
| Recognizing the contribution of PLWHIV to HIV cure-related research | FG-1 | Participant | *[S]omeplace where people could actually go and have some closure, for lack of a better term … And I think we may be offering that opportunity to anybody who’s in the study, if they want their picture or, you know, they wanna post their story or something like that, you know? Allowing them to do that and having a place, you know, kind of a memorial wall or something.* |
|  | FG-1 | Participant | *And it would be something lasting that those who are altruistic enough to participate in [HIV cure-related research], get something somewhat everlasting and gives them some comfort for going through all this.* |
| **Ensuring Social Acceptability of HIV Cure-Related Research at the EOL** | | | |
|  | 102 | Bioethicist | *So the goal here is to hear from the community, to engage the community and not get ahead of them.* |
|  | 104 | Community member | *Branching out into the community and letting them know that this kind of cure research is happening I mean it's part of the continuum.* |
|  | 104 | Community member | *I think continuing that process of getting the word out into the broader community and not just in the kind of close-knit HIV advocacy and patient, um, advocacy groups is important.* |
|  | 114 | HIV clinician | *Communication, again, especially in this day and age. I will not touch on what's happening in the larger world at this time. But it's obvious that research practices, as they happen, happen in the insular academic world. But when the message needs to be communicated to the larger community at large, oftentimes, that message is lost. The general population can hear something distinctly different from what the researchers are trying to say. Communication, I think is very important. I think frequent and concise and simple communication.* |
|  | 114 | HIV clinician | *Be very upfront about what they're trying to do with the study. Be very upfront that, it is of no more harm to the participant than would be otherwise. These concepts need to be communicated very clearly.* |
|  | 105 | Researcher | *I think it's reasonable to say we went to the community that matters the most, people with this disease, and that community feels comfortable with us proceeding…. It needs to be well implemented and have clear outcomes that will help the affected community.* |
|  | 107 | Researcher | *I think one thing we've learned just from everything that's happened recently with COVID is that people find out their news and create their judgment based on social media and not science and their peers and their friends and not science. And that's a generalization, but I think we really try as scientists to make papers, to like have more data to kind of support our theories but I think it's really going to take more...* |
|  | 107 | Researcher | *So just, I think getting more creative on how to reach populations outside of the HIV population.* |
|  | 108 | Researcher | *I think that the public needs to know what type of research we're doing. We need to explain it to them, we need to ask for their input, their voice on how they interpret what we're doing. And then when we receive that information, we need to respond to it because oftentimes what a scientist has in mind for how they wanted to describe a study, isn't necessarily the same information that the participant receives. So we need to make sure that we let them know what is safe, what has some risk, and what that risk may entail and if there's no risk* |
|  | 119 | Researcher | *I think continuing to have public discussion and dialogues not only about the pros and cons of the studies that we're doing, but the alternatives. Are there better alternatives? If there are, should we be considering them? … Is there equipoise both in the patient, public community and in the scientific community for what we're doing?* |
| **Navigating Potential Conflicts between Research Aims and Clinical Care** | | | |
| Recognizing the patient/participant’s decision has ultimate priority | 102 | Bioethicist | *So it’s first the patient’s decision.* |
|  | 104 | Community | *I think at the end of life, there really isn’t much conflict.* |
|  | 104 | Community | *I think the most important part is to really have an in-depth conversation with the patient to find out what their desires are…. So, I think it really comes down to what the patient wants and how much they're willing to do and how much they wanna participate.* |
|  | FG-1 | Participant | *I would think that if you're gonna err you wanna err on the side of the patient … you don't interfere with the patient.* |
|  | FG-3 | Participant | *I would say the simple answer is the patient.* |
|  | 111 | HIV clinician | *So I think that the participant decides which one that they prefer. I think as long as we give our participants a choice, and at any time to opt out, so if they say yes and then at some point they're like, "Oh wait this is way more than what I was expected, I can't do this anymore," we respect that and stop, then I think that that's what you got to go with. Some of those potential conflicts should probably be addressed at the very beginning in recruitment.* |
|  | 120 | HIV clinician | *As far as I'm concerned, the patient is the one who makes the final decision. I tell them that, really, they are the ones who are in charge of their decisions. I'm a very good navigator, but it's their boat.* |
|  | 120 | HIV clinician | *Well, I think the patient would be the ultimate decision-maker. Gosh, I guess it would be best if everyone was on board the same way, but it's similar to hospice care in a way.* |
|  | 101 | Researcher | *The patient. Patient/participant. Their role is not to be cared for, and they must be the consenting individual.* |
|  | 106 | Researcher | *I think if there's a conflict, ultimately, the patient has to make a decision. It all depends on the details, but basically, presumably, if the patient's deciding to do it, it has to be a three-way decision, I would think. In most cases, the care provider... The participant informs them what they want to do, and unless the care provider objects, that's fine.* |
|  | 108 | Researcher | *A conflict, I guess it would depend on what kind of conflict it was, because if it's a conflict of, do I want to participate? I don't want to participate, that's all in the participant's hands.* |
|  | 108 | Researcher | *if I was to make an error, I would make the err on the side of the participant, if they're able to give that information, if they're not, then whoever they put in to make the decisions for them in their absence would be the person who would be making that decision.* |
|  | 108 | Researcher | *It's always going to be the participant first. To me, the hierarchy is always going to be the participant, their significant others, and then their providers, and then research is right down here on the pyramid.* |
|  | 110 | Researcher | *Cases are going to be few and it's going to be a challenge to have enough numbers. I don't see a tension between the research side and the clinical side.* |
| Recognizing clinical care takes precedence over the research aims in the absence of a contrary participant’s decision | 114 | HIV clinician | *I think that, if there ever is a conflict that the study participants needs, clinical needs should be first and foremost.* |
|  | 115 | HIV clinician | *Clinical care to me would always win out.* |
|  | 105 | Researcher | *And I think the biggest thing to keep in mind is whatever clinical care needs to happen is the priority. And if a study visit needs to be canceled because somebody has a clinical test or something like that has to happen, that should be the first priority. It almost should be like a consultant relationship. It should be a recommendation made to the clinical team… but let the clinical team always make the final decision* |
| Ensuring communication between the research team, the participant, and the participant’s clinical care provider | 114 | HIV clinician | *Again, communication is key with study staffs, the study coordinators, participant caregivers and the investigators.* |
|  | 115 | HIV clinician | *I think just for me communication with providers, especially, I think providers who are involved is probably important, at least letting people know what's going on and really very clearly to the patient knowing where the division of labor is, as best as that can occur.* |
|  | 115 | HIV clinician | *And I didn't think there was great communication about that. And that's what I worry sometimes with studies like this, especially if they're intensive interventions, and the patient has any confusion over what's his clinical care, and what's his research care, and does that get blurred in some way?* |
|  | 120 | HIV clinician | *I think in that case, though, it would be more of a communication between the researcher and the clinician.* |
| Ensuring decisions are made on a case-by-case basis | 102 | Bioethicist | *[B]ecause everyone is individual, I’d say on a continuum.* |
|  | 116 | Researcher | *They're all tough questions. I think a team approach is my only simple answer to that … having somebody independent of the protocol who is at least advising is available.* |
|  | 119 | Researcher | *The decision may be different from day to day. So, I think this is a moving field, and it needs to constantly evolve. Depending on who's in the room, the outcome is going to change. Again, I think mostly the answer is always having the voices of the people who are involved in the research front and center is the most important.* |
|  | 119 | Researcher | *[The] idea of deliberative democracy. So, I always think we need to have continued discussions and dialogues, but I thought this might be one of those situations where if you brought participants and experts in the field and just community members into a room all together and had this deliberative democracy where you had this idea where you have both elements of consensus decision making and majority rule and had that informed decision. Again, I'm not saying we would need to follow the rule that came about, but it would be an interesting way of approaching this who gets to decide, particularly as we move forward with potentially more invasive approaches to see if there was in fact a consensus around a large group of people that came at this from somewhat different, you know, people at the end of life, people who are nowhere close to the end of life, and scientists across kind of a range of different disciplines. It would be interesting to see if there was a consensus at the end of the day for one consensus opinion.* |
| **Role of Advance Directives in HIV Cure-Related Research at the EOL** | | | |
| Support for advance directives in HIV cure-related research at the EOL | 104 | Community | *I think it's key. You really need to encourage people to do that and think through all the scenarios.* |
|  | FG-1 | Participant | *It eliminates confusion, it eliminates, conflict within the family ... like as I say eliminate any potential legal problems, issues with the family members within the family.* |
|  | FG-1 | Participant | *And so you can refer to that document and the family, hopefully, will get some comfort out of knowing that these are their wishes and this is how they wanted it to be even though it looks intrusive or uncomfortable to them.* |
|  | FG-2 | Participant | *I would just say having really clear advanced directives and also making sure that the participant's had conversations with his family members and loved ones, saying, "This is what I want. This is for me and please don't interrupt, interfere with my wishes."* |
|  | 105 | Researcher | *So people need to have the opportunity to frequently in an ongoing basis review their end of life goals or advance plan care planning.* |
|  | 108 | Researcher | *And I've done a lot of this work out in the community talking about advanced healthcare directives, because it's something that's just a great public health need, whether you have HIV/AIDS or any other chronic condition. And everybody 18 and over should be filling out one of these advanced care directives. It is so very important. It fits at the end-of-life because at some point in time, the individual is going to be potentially unconscious, not everyone has a swift death, some people remain in a semi-comatose or comatose condition for several hours to a few days prior to their death.* |
|  | 115 | HIV clinician | *But on an advanced directive to me, it'd be more about what research could look like, should you no longer be able to agree to anything.* |
|  | 108 | Researcher | *It's something that I would love for each individual to have, an advanced care directive, describing how they like their palliative care to be directed as well as how they want their final moments to be directed. However, it's very individual. Some are very willing to do this and some it's a very difficult process to deal with.* |
|  | 118 | Researcher | *Well, in essence, the whole enrollment into the Last Gift cohort is an advance directive. I think it should be a requirement to enter such a trial … that you should have an advance directive, as an eligibility criteria.* |
|  | 119 | Researcher | *My answer was as a service to all participants, we should be encouraging our participants to have an advance directive.* |
| Ensuring initial discussion of advance directives are with a patient’s primary/clinical care provider | 105 | Researcher | *So, I feel like an advanced directive is really to be discussed with the primary care provider or the team…. I think that's often the case, that seed should be from the standpoint of the professional who knows that person the best.* |
|  | 105 | Researcher | *But I think the goal needs to be ... and then that conversation happens between the clinical provider and the patient. And then the same conversation or similar conversation should happen between the research team at enrollment. What are your goals? Can you confirm to me what you've already told the clinical side? What would you want us to do if you reach this point or that point? Would you want us to step back* |
|  | 105 | Researcher | *But maybe it is a go no go question. "Have you had a conversation about your advanced care? Yes, you have. Great. We would like to confirm those with you now. And we can compare this document to what you've signed with your other doctor." If you say, "No, I've never talked about it." You say, "Okay, we're stopping here. You still consented. But we want you in the next month to have a conversation with your doctor about your advanced care planning, and then come back to us and we'll confirm those decisions."* |
|  | 107 | Researcher | *But it makes a lot of sense to me that there's some form of documentation whether it's one of the inclusion criteria is that they provide an advanced directive so rather than you complete it, maybe just have a copy of it. So you know that they've discussed it with their provider.* |
| **Role of Palliative Care in HIV Cure-Related Research at the EOL** | | | |
|  | 114 | HIV clinician | *They don't necessarily have to be at odds with one another. I think palliative care can continue as a research study is continuing. I think it's similar to what I was saying earlier about, informing the palliative care team about the research study and the research study should also be aware of the palliative care team.* |
|  | 105 | Researcher | *I don't think researchers should be afraid of palliative care doctors and I absolutely think any participant who's really going to be spending a lot of time considering end of life and considering symptoms and potentially even more suffering then might naturally happen because of a research study or something like that. Then yeah, they should absolutely be engaged with palliative care.* |
|  | 107 | Researcher | *They might have chronic pain.* |
|  | 108 | Researcher | *Well, it's really important to have pain control. It's important to find out what the participant wants. How much pain control do they want? What type of feeding do they want? What type of oxygen, if any, do they want? What type of IVs, if anything, do they want? They're the ones that are going to make that decision. And often times it's appreciated because a lot of people assume, you'd be surprised, a lot of people assume that it's going to be taken care of, but it really needs to be directed, and it needs to be directed by the participant and their next of kin, the people who are on those advanced care directives.* |
|  | 116 | Researcher | *Should be hand in glove. I think they have to be in some way a part of the protocol. Especially if you're testing a new intervention, you need somebody outside to look over those issues … so having somebody with palliative care would be quite useful. I don't know if they need to be on the research team but they have to be a part of the care team.* |
| ***Additional Considerations for HIV-Cure Related Research at the EOL*** | | | |
| **Role of HIV Care Provider in EOL Translational Research** | | | |
| Acknowledging the close relationship between PWH and their HIV care providers | 111 | HIV clinician | *HIV providers are unique. We're not your typical primary care doctor. … So many of us see ourselves as advocates. So we're not just a provider but we're an advocate, and some of us even view ourselves as extended family.* |
|  | 111 | HIV clinician | *I do think that, particularly at the end of life, providers tend to become very protective. Especially because we don't want to see them [patients] suffer. Some people have been around since the 80s and 90s and [we] really don't want to see that again. And I think that can be difficult.* |
|  | 114 | HIV clinician | *The HIV care provider can play a significant role… But I think in most cases and certainly is the case, I think in my experience at my clinic, patient and provider relationship is quite significant. Because we tend to know each other over many years, see each other over many years closely.* |
|  | 114 | HIV clinician | *Sometimes, patients, individuals, will be hesitant to participate in research studies without a seal of approval, so to speak, from their provider. Making sure that there is a blessing, even cursory, that the provider at least reviews what the research study is talking about and can say confidently that it's, first of all, that it's safe and second of all, that it's in line.* |
|  | 115 | HIV clinician | *There were certainly a lot more calls, hospitalizations, things I needed to do and were more involved in their care in the end of life.* |
|  | 115 | HIV clinician | *I think there are some providers who would really like to potentially be part of the process, whether that's just sort of getting updates about here's where we are. And maybe it's something that is part of the consenting process for the patient where the patient decides, "I'd like my provider to be aware of what's going on."* |
|  | 119 | Researcher | *A lot of these folks are very attached to their primary care provider and they want to know that that person agrees with their decision if it's important to them.* |
| Ensuring open communication between research teams and HIV care providers | 111 | HIV clinician | *I mean certainly researchers don't know how to talk to clinicians. I mean any time you go to any kind of conference and you hear a basic science researcher, or a translational researcher share their data, clinicians don't know what all this stuff is. So if you can't even speak... if you're not even willing to take the time to speak the language, and really sit down with people and talk with them about why this study is important, and how their patients could benefit, you're never going to have them be on board for it. And all they're going to see is the perceived risks.* |
|  | 120 | HIV clinician | *If they [the researchers] were able to keep the clinician as informed of what's going on, that would be really, really good* |
|  | 101 | Researcher | *Well, I think one of the more important roles is for the provider to provide the researchers with information that might be pertinent to the likelihood that a readout could be obtained before the patient dies. For example, any disease burden that might alter the risk of the treatment. And those would be the main things. Yeah. If a participant was interested in having their primary care provider involved, which I think is likely to happen at times, I think it's entirely appropriate for there to be consultation with them, with that person, and that might be very reassuring to the participant.* |
|  | 108 | Researcher | *“Please let your primary care know that you are participating in this study." So it's going to be really important that they're aware of what's going on.* |
|  | 108 | Researcher | *[A] lot of physicians and in turn a lot of families were not aware of the possibility of organ donation for folks who are HIV positive. So that is still a struggle today. Even with the Last Gift, we try and try and try, and there is a lot of turnover in providers, HIV providers, other types of providers like oncologists. … We also could do that with providers. I set up in-services with providers at the VA or at various clinics to let them know what we're doing, what we've done so far, what the results have been and what we're planning to do. And we ask them for their input.* |
|  | 113 | Researcher | *I think that's always a good idea to involve all the people who are important people in a person's life and care. So involving them is always the right thing to do, as it is if it’s a courtesy, in any research participant, to let the providers know that patients are participating, … So the more communication, the better.* |
| **Relevance of EOL Translational Research to Other Fields** | | | |
|  | 104 | Community member | *Definitely cancer. And I would hope that this would kind of pave the way for this kind of research in other fields, so we could benefit from it.* |
|  | 104 | Community member | *So yeah, any kind of research, you know, COVID, whatever, that we could use, apply this for, so I think with the only benefits, you know, science and humanity.* |
|  | 111 | HIV clinician | *Particularly like Hep B … but I do wonder if, in other conditions like advanced liver disease from hepatitis B or cancer, if they're going to see that these interventions as another hope to live longer. … I do wonder, if you're having another intervention in another condition, whether or not it starts to become coercive because they start thinking that maybe this could then extend life.* |
|  | 114 | HIV clinician | *It could definitely inform other fields such as cancer related research.* |
|  | 120 | HIV clinician | *Parkinson's. What is it? Lou Gehrig's, ALS, all sorts of ones.* |
|  | 101 | Researcher | *Sure. I'll give you a, I'm a neurologist, give you an example may be relevant to my field. So, ALS is a devastating, progressive, relentlessly progressive, neurological illness that kills people.* |
|  | 116 | Researcher | *Yeah, well I guess it's come from the cancer fields. I think it's also relevant. hepatitis B, I guess.* |
|  | 117 | Researcher | *Hep B, that might be actually for all these persisting viruses that establish reservoirs; Hep B is one of them actually. You might actually be able to understand better about persistence by doing this type of research.* |
|  | 119 | Researcher | *I think sometimes, too, of rarer cancers. If you have a rare cancer, and you have a group of people who are at end of life, it would take forever. You probably can't ever do it to study novel drugs, but if you had five people who are at end of life, you could probably figure out whether a novel drug worked in that group of people.* |
| **Cultural Considerations in EOL HIV Cure-Related Research** | | | |
| Understanding culture as determinative of participants’ engagement and their communities’ acceptance of EOL translational research | FG-2 | Participant | *[U]nderstanding the cultural background that the patient comes from. That's key because if there are certain traditions and expectations of when a loved one passes away.* |
|  | FG-3 | Participant | *I think everybody has their own experience, their own culture, their own things that they take with them. And we're all a little bit different.* |
|  | 111 | HIV clinician | *I think there's a lot of different ideas about death, in different cultures, and presentation after death, so whether or not an intervention is going to impact the body… All of those things I think could certainly come into play.* |
|  | 120 | HIV clinician | *Well, I don't know how one would separate the person from the culture, but I absolutely think it would play a big difference.* |
|  | 119 | Researcher | *I think they play huge differences both from the role of the scientist. I think there are probably scientists that don't approve of this at all based on their religious, ethnic, whatever backgrounds and probably even more so from the participant side of things.* |
| Recognizing individualistic versus collectivist societal views | 102 | Bioethicist | *There are other societies in the world, other cultures, and many of those people live in this country who put much more weight on what the family or the group believes is appropriate rather than the individual. And for them, they would be appalled at the idea that the individual would make this decision by herself or by himself.* |
|  | 111 | HIV clinician | *[T]here are differences in individualistic versus collectivist type societies. So, a person may take into consideration more the impact of what's going on with them to their family, rather than to themself if they're part of a more collectivist type family unit, rather than a more individualistic type unit.* |
|  | 103 | Researcher | *I can see this being a problem for certain culture that they value their body. … And some people don't believe in science, right? And so why should they contribute to research? I can totally see how they might not like it if we take any final minutes away from them and their next of kin.* |
| Appreciating differences in how death is perceived across cultures | FG-3 | Participant | *[O]ne thing is people view death differently. We're looking at it from a western industrialized nation perspective. Those from other countries don't have the same phobias and fears that say, we do here in the US.* |
| Recognizing religion as an important cultural consideration | FG-1 | Participant | *I think that there're religious components to that. You know, if someone's Catholic, if someone's Jewish, if they're, you know, if they're Jewish or Muslim and the culture is to have the body buried intact within 24 hours.* |
|  | FG-3 | Participant | *One thing that came to mind for me was just religious cultural differences and just having family members onboard because... Is the remains of the participant returned to them from a crematorium?* |
|  | 114 | HIV clinician | *Especially Hispanic and Latino cultures that are very religious would not be, I think, comfortable with the idea of anything hastening death. Death is a natural event. In their beliefs, nothing should hasten it.* |
| Recognizing medical mistrust as a significant cultural barrier to be overcome | 104 | Community member | *You know the African American community is a classic example, right? With all the medical mistrust for obvious reasons. … And I think when engaging those communities, you have really have to address their kind of more urgent primary concerns. I mean access to treatment in the first place and all the other kind of social determinants of health, if they're facing what the co, you know, end up killing them, right? I think finding a way to address those in a caring and compassionate way would go a long way.* |
|  | FG-2 | Participant | *And I think we need to keep in mind that there's ... In a lot of communities, there's medical mistrust for very good reasons because there's been really examples of that in the past. Like Tuskegee and Henrietta Lacks.* |
|  | FG-2 | Participant | *I think in a larger perspective, just getting the study team [to] work on getting the word out to those communities, making alliances with… communities of color, African-American[s], and Latinx. Um, just to get out there, work with trusted community leaders to say, "This is what we're doing." Maybe have people who have been involved in the research talk about it, so it demystifies it and makes it a little less scary and helps bring down some of the barriers of past medical mistrust.* |
| **COVID-19 and Rapid Research Autopsies** | | | |
| Determining cause of death to ensure the safety of the autopsy team | 104 | Community | *We'll figure out a way to ensure the safety of the autopsy team. They already have universal precautions, right?* |
|  | 111 | HIV clinician | *[S]o how it affects it is that health care providers don't want to do anything that's not necessary, because they don't want to put themself at risk . … I do think that we don't know if COVID can be spread with handling of tissues during autopsy. I doubt it, but we don't really know. So I could see why maybe the pathologist would not be willing to perform an autopsy if it's not medically necessary.* |
|  | 103 | Researcher | *I think that COVID definitely affected our rapid autopsy research program because during the autopsy we perform, there are aerosols. And so there is an increased risk of infection if the actual patient is COVID positive, this is why we test every patient before we perform an autopsy for COVID. And if we get COVID positive patient, we will have to use special equipment and protection for the rapid autopsy team.* |
|  | 106 | Researcher | *It just requires additional precautions.* |
|  | 119 | Researcher | *There's a lot of aerosolization, so I think in today's day and age, you should worry about whether you should be doing autopsies if you can't test for COVID in the 20 minute turnaround time, because you don't know. … I think the only safe way to do it is to have the ability to check for COVID with an immediate turnaround time to know that your staff are safe.* |
|  | 119 | Researcher | *Well, I can tell you from our side if it's not perceptions, it's practice. Now when someone dies, the question is we don't always know why they died. Did they die with COVID? And so, we have to take very different autopsy precautions if the participant had COVID, and since we don't know, we now have to test everybody for COVID before we're allowed into the autopsy suite. So, that delays us.* |
| Acknowledging that COVID-19 has affected dying rituals | FG-2 | Participant | *I've seen effects of people not being able to have the funerals or having a closed casket and not be able to touch the body.* |
|  | 120 | HIV clinician | *I think that because COVID has illustrated health disparities so clearly and has also helped us to recognize that dying alone is not the way that most people want to do it.* |
| Recognizing the scientific disbelief and medical mistrust of COVID-19 | 120 | HIV clinician | *I have to say I'm a little bit disheartened by how many people don't appear to be concerned about COVID.* |
|  | 107 | Researcher | *I think that's a very polarizing effect of COVID on research [because] so many people don't really believe in COVID.* |
| ***Perceptions of Medical-Assistance-in-Dying (MAiD)*** | | | |
| Recognizing a patient’s autonomy in choosing MAiD | 104 | Community member | *[L]etting people kind of die on their own terms … so that they can be allowed to die peacefully and not linger, which would be incredibly painful.* |
|  | FG-2 | Participant | *When the patient decides, "This is when I'm going to end it," that gives them, you know, a chance to kind of prepare.* |
|  | 114 | HIV clinician | *I actually don't see much of a conflict there at all. In fact, the fact that California now has assisted death or end of life, that means that patients, by precedent, now have greater autonomy over what to do at the end of life. In fact, I think it would go along nicely with end-of-life research in possibly high risk interventions at the end of life. Because it would fall in line with the type of autonomy that is now acknowledged in states like California at the end of life.* |
|  | 103 | Researcher | *I think it's a personal decision and as long as patients know what they're doing and they go through the proper channels and processes, I am totally in favor of this and I don't think it's mutually exclusive with [research at the EOL]. I think it's actually a perfect combination as long as it's done ethically.* |
|  | 108 | Researcher | *That's something where the participant is at the top of the pyramid. The participant makes that decision of when that's going to occur, how that process is going to take place and we follow them.* |
|  | 108 | Researcher | *There are already state mandated processes and protections in place for when someone decides to take advantage of that prescription and end-of-life process. We follow what the participant wants to do, and we work with that and around that to make their wishes happen.* |
|  | 109 | Researcher | *Medical aid in dying allows people more autonomy on their death and how they want to go about in their own terms.* |
|  | 110 | Researcher | *[I]t's a matter to make sure that they're taking this decision independently that everything will be done in a way that respect their wishes.* |
| Separating the MAiD decision from the research | 104 | Community member | *It should be a completely separate discussion.* |
|  | 120 | HIV clinician | *I think you'd have to be really clear to separate, in this case, the clinician from the researcher because you want to be able to very much trust the purity of the patient's decision.* |
|  | 103 | Researcher | *I think that the person testing the intervention and the person providing the end of dying paperwork should not be the same. … People should coordinate the two processes, but it should be two different people, two different and independent people to provide the two services.* |
|  | 106 | Researcher | *I think it would be a wise for end of life investigators to try to not get their studies confounded with the act, to some extent for public relations or publicity issues.* |
|  | 112 | Researcher | *Yeah. So it's a really, you have to have two separate entities or physicians, probably entities do analysis for the intervention.* |
|  | 116 | Researcher | *I think in the United States there's the added issue that you're being supported by federal funding. So that has to be clearly separate from what the patients do.* |
|  | 119 | Researcher | *We're doing a research study, and I think the medical aid in dying is a primary care issue, and I think the primary care physician should care for that.* |
| Need for honest communications, mental health evaluations and independent reviews in decisions to undergo MAiD | 111 | HIV clinician | *I don't think it's wrong to have a safeguard... kind of an oversight committee like we talked about, planned for these studies.* |
|  | 109 | Researcher | *It's not necessarily that it excludes them from the research, but we have to have the open and honest discussion about what their goals are both on the aid in dying side and on the research portion.* |
|  | 113 | Researcher | *I think that's where I think really paying special attention to mental health is important and making sure that people are evaluated for depression and offered treatment for depression if it exists.* |
